# Our Vienna Letter

**Published:** 1891-08

**Authors:** Bransford Lewis

**Affiliations:** Vienna


### FOREIGN CORRESPONDENCE.

### OUR VIENNA LETTER.

VIENNA, July 6, 1891.

*To the Editor of the Southern Medical Record:*

DEAR SIR—As the methods of pursuing one's studies in Vienna are quite different from those in vogue at home, it may be of interest to some of the readers of the Record to learn something of them, so that they may get to work with as little loss of time as possible, after arriving here. There are two kinds of courses, those given by the "private docents" (private instructors), who are assistants to the professors, and those given by the professors. The latter are continued throughout the two sessions, viz.: from October till Easter and from Easter till the middle of July. The courses of the docents last from four to six weeks ;they have no fixed time of beginning or ending, but generally as soon as one such course is finished, another is begun, with a new class of students. These number from eight to twenty, according to the popularity of the teacher. The docents, who are authorized by the University to teach in this way, obtain their clinical material from the clinics of the professors whom they assist, either out or in patients being used for the purpose. Some docents, as for instance, Dr. Finger, have their own clinics.

The cost of each course is from 15 to 20 guilders (about $7.00 to $10.00) ; operating courses and those in obstetrics are higher, 50 guilders ($25.00.) With graduates, Americans, for instance, who come to study in branches, the courses of the docents are much preferred to those of the professors, generally, as there are fewer students in them, and closer access for examination is had to the patients, questions may be asked of the teachers in the midst of his remarks, etc.

It is thus seen that teaching is going on all the time here, so that one need not be afraid of arriving out of season. The season for the docents lasts the entire year.

One of the greatest advantages offered the post-graduate students here, is the opportunity for hearing the views of different men on the same subject, and at the same time putting in as much time as he likes on any branch. Thus on syphilis, one may in a single day hear Prof. Kaposi, Prof. Lang, Prof. Neumann, Drs. Finger, Ehrmann, Zisol; Riehl and Cehak, each demonstrating his remarks with selected clinical cases; and to hear these several teachers, one need not go outside of the Trankenhaus, and therefore loses no time going from one to another. I believe that in this respect Vienna stands alone; and to the student who has only a short time at his disposal, this is a point of the utmost importance. Americans doubtless appreciate this most; for they are without doubt the busiest and most time-economizing people on the globe.

But it requires an enormous supply of American energy to keep one going in true American style after he lives here a short time; for the blandishments of Vienna life are truly overpowering!

When on a bright and sunny holiday, in celebration of the birth—or perhaps the death?—of some old grandee whom everybody has forgotten, but whom everyone continues to honor with a genuine holiday (they wouldn't lose a holiday out of the year here for anything, although I believe they have about seventy-five)—when, I say, on such an occasion, one feels that all Vienna in its Sunday attire has gone out to Prater to promenade or drive up and down the broad and beautifully shaded avenues, or sit in the cafe gardens laughing, chatting, drinking in the sweetest of music, as well as more substantial refreshment, he doesn't generally keep up work on his dictionary and his scientific and tough German medical book very long that day, but gets up and adds himself to the thousands of frolickers that have gone before.

Among the most interesting clinics in Vienna is that of Prof. Kaposi, and the professor himself is one of the most popular teachers here. Rare cases of skin diseases, which we have only read about, are continually turning up there. I doubt if many of your readers have seen a *plica polonica*, a condition of the hair, in which, from schorrhoea, neglect, pediculi capitis and filth, all combined, it is matted together in a mass that resembles a discarded weaver's net; or corcinoma growing from a lupus ulcer on the face; or sarcomatous change of the entire skin of the body; or a rhinoscleroma, in which the nose has undergone indurating changes that make it feel as though it were carved out of wood; or the condition called argyria, in which, from prolonged or excessive ingestion of nitrate of silver, the skin (more especially that of the face) takes on a steel gray or silvery hue that makes one wonder whether the patient is really of flesh and blood, or is an animated silver statue. Examples of such conditions, as well as of many rare manifestations of syphilis, I have been fortunate enough to see in Kaposi's clinic during my short stay here. He also showed a case of smallpox in the amphitheatre one day, and at another time, two girls with leprosy.

He, or she, who enters this clinic as a patient, must leave modesty behind; for the cutaneous surface of every patient is shown with a completeness that leaves no doubt in the mind of the investigator as to whether he has seen all of the lesions or not. Though female nurses are in attendance, male patients are stripped entirely; and females usually disrobe as low as the waist, and then, if necessary, as high as the waist. In Vienna, the cause of science must not be sacrificed to the cause of proudey. In the venereal clinics the female nurses usually apply the dressings to chancres, chancroids, etc., on the genitals of either sex. In Dr. Grunfield's endoscopic clinic, the nurse is frequently able to give a friendly pointer to the timid student as to size of tube appropriate to individual cases.

A feature in connection with Kaposi's course, which is of great assistance to him and equally advantageous to his students, is his seemingly inexhaustible supply of lithographs of all kinds of affections of the skin. Having inherited, I suppose, a great number of these from his father-in-law, Hebra, he has been continually adding to them, so that now his collection is valuable in the extreme

With respect, I am, etc.

BRANSFORD LEWIS, M. D.

---

The following is said to have happened in a certain Bible class at a very noted female college in Virginia:

Lady Teacher—Miss Annie, what do you understand by the word "circumcision" used in to-day's lesson?

Miss A.—It is taking the scalp off of a male baby when eight days old.

